# Bilateral upper limb amputations in victims of high tension electrical injuries: Three case studies

**DOI:** 10.4102/ajod.v4i1.117

**Published:** 2015-08-27

**Authors:** Cajetan Nwadinigwe, Obidiche S. Olewe

**Affiliations:** 1 National Orthopaedic Hospital, Enugu, Nigeria; 2 Department of Orthopaedics, National Orthopaedic Hospital, Enugu, Nigeria

## Abstract

Bilateral upper limb amputations result in severe disability. High voltage electrical injury is a rare cause of such an outcome and injuries often occur as occupational hazards. We present three case reports of accidental high voltage injuries that occurred in a non-occupational setting. Victims were all initially managed at other centres before referral to our hospital and all subsequently had bilateral upper limb amputations. The high cost of treatment, importance of prevention, and need for rehabilitation are highlighted.

## Introduction

High voltage electrical injuries are caused by exposure to voltages equal to or greater than 1000 V (Escudero-Nafs *et al.* 1990) These injuries are relatively rare (Okpara *et al.* 2006) when compared with other causes of burns presented to the emergency units and especially so in people who do not routinely work on overhead high voltage lines. For those who survive such injuries the long term sequelae can be devastating. Apart from cutaneous injuries, there is massive destruction of underlying muscles, nerves, blood vessels and bones which often requires amputations (Escudero-Nafs *et al.* 1990). Studies show that up to 49.4% of victims of high voltage injuries would need amputations (Hussmann *et al.* 1995) with the majority in the upper extremities because the hand is the usual primary point of contact (LaBorde & Meier 1987; Remensnyder 1980).

The single most important factor related to risk of amputation appears to be the voltage strength (Oluwatosin 2004). Mortality in the acute period is often as a result of cardiac and respiratory arrest, shock, renal failure and sepsis and has been reported as between 0% to 18% (Janjua 2002; Remensnyder 1980). Regional studies indicate that victims are often electricity company workers and vandals (Abbas *et al.* 2009). This contrasts with non-electricity line workers who routinely do not have to deal with high voltage lines but may become accidentally injured as is seen in the cases presented. Less than 6% of victims are eventually able to return to their previous line of work (Hussmann *et al.* 1995). Prolonged hospital stay, multiple surgeries, long rehabilitation processes and heavy financial involvements are attendant issues.

We present case reports of three young men who suffered severe high voltage injuries necessitating bilateral upper limb amputations.

### Ethical considerations

Ethical clearance for this research was approved by the National Orthopaedic Hospital, Enugu, Department of Orthopaedics (Nigeria); IRB/IIEC number S/313/855, protocol number 156.

## Case report 1

Mr S.H. was a 28 year old self employed welder who was referred to the National Orthopaedic Hospital, Enugu (NOHE), from another tertiary centre after a high voltage electricity injury sustained one week earlier.

The patient had accidentally touched an overhead high voltage wire (supposedly transmitting 11 000 V) with a long pole whilst working on top of a one storey building situated under a high voltage line. He was wearing gloves but was not wet at the time of the incident. Following contact he was transfixed to the pole for about 20 minutes before being rescued by a neighbour using a wooden stick. He lost consciousness but did not fall from the building. He was taken immediately to a nearby teaching hospital where he regained consciousness after about an hour. Severe burn injuries to all his limbs were identified. Following initial treatment, he was referred to our centre at his request after he declined an offer of amputation. We found gangrene of the left forearm extending up to the cubital fossa, and gangrene of the right upper limb from the hand to the proximal third of the forearm with exposure of the radius and ulnar. The right lower limb had 14% septic deep dermal burns with extensive eschar. The left lower limb had 4% mixed thickness burns with eschar on the dorsum of the foot.

**FIGURE 1:**
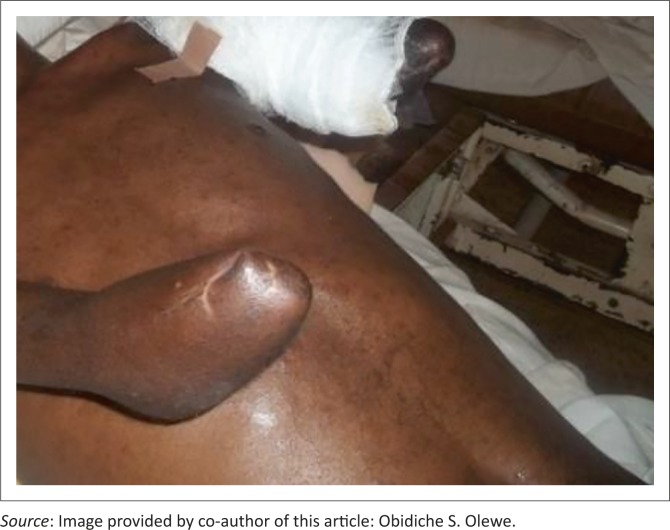
Mr S.H., three months after bilateral upper amputation.

The patient was co-managed by the plastic and orthopedic surgery units. He was properly counselled and had provisional bilateral amputations of the upper limbs the same day (left above elbow and right below elbow) as well as wound debridement and escharectomy of the lower limb wounds. His rehabilitation is ongoing at the time of this report (Figure 1).

## Case reports 2 and 3

Masters A.C. and D.E. are cousins who sustained severe high voltage electrical injury whilst trying to adjust their television antennae to obtain a clearer view during a world soccer tournament. The TV antenna accidentally touched a high voltage electric wire passing directly over their roof (11 000 V transmission line). They were transfixed for a length of time until they were extricated using a wooden stick and then transferred to a nearby general hospital where they were initially treated before referral to our centre by the fourth day.

**FIGURE 2:**
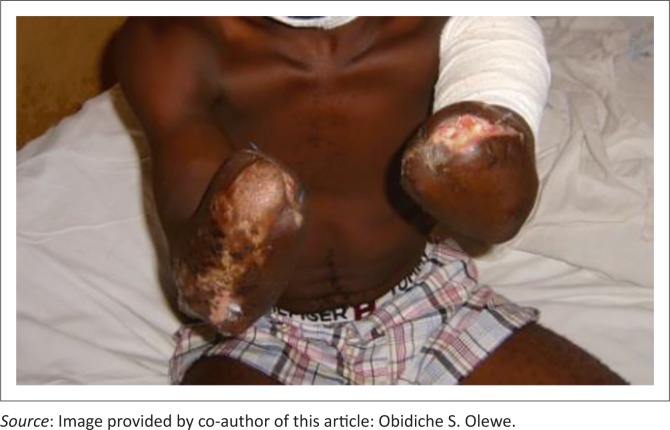
Mr A.C., five weeks after amputations.

**FIGURE 3:**
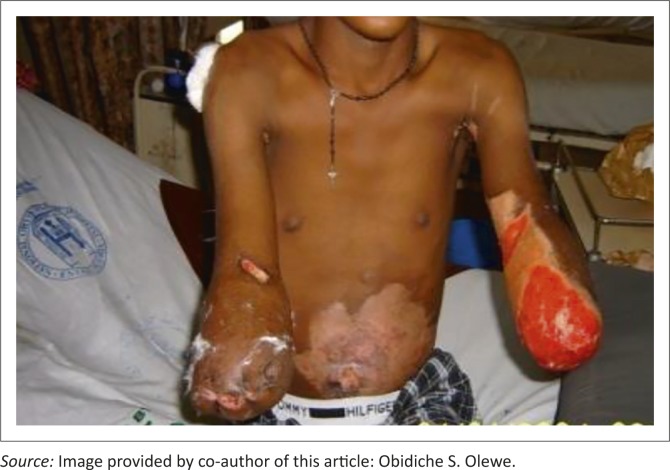
Mr D.E., five weeks after amputations.

On presentation, both had gangrenous upper limbs up to the mid forearms. D.E. had, in addition, flame burns of the anterior abdominal wall, left medial distal arm and the axilla totalling 10% of the total body surface area (TBSA). Patient A.C. had, in addition, flame burns of the left arm, posterior aspect of the right arm and temporal aspect of the scalp, totalling 8% TBSA; he also lost his left ear. All the burnt surface areas were infected. Their 24 hours urine output was greater than 1.8 L per person. They were counselled for amputation to which they consented, and bilateral below elbow provisional amputations were performed and later re-fashioned (Figure 2 and Figure 3).

Upon discharge from the hospital there was no follow-up.

## Discussion

High voltage electrical injuries are caused by exposure to voltages equal to or greater than 1000 V (Escudero-Nafs *et al.* 1990).

Passage of current through tissues leads to electrothermal heating, generating temperatures of up to 4000°C and more. This can result in extensive tissue damage along the path of current flow. Theoretically the heat generated may be determined from the formula GC = C2R/4.187, where GC is the heat in gram calories per second, C is the current in amperes and R the resistance in ohms (Knight 2004). Typically there is a ‘source’ and ‘grounding’ wound corresponding to the points of contact and exit of currents from the body. Grounding injuries may be multiple. In the three patients presented, the source of current was through both hands whilst grounding was via the lower limbs. The extent of cutaneous injuries is often just the tip of the iceberg compared to the depth and extent of underlying tissue damage. Other mechanisms of injury include flame burns, arc injuries, conduction abnormalities of the heart, as well as secondary injuries from violent muscle spasms and falls. Mortality is usually because of cardiac and respiratory arrests, shock, renal failure from myoglobinuria and sepsis.

Treatment must be prompt, with aggressive resuscitation, cardiac monitoring, organ support, wound care as well as supportive care (Arnoldo, Klein & Eubran 2006). Survivors often end up with amputations (Escudero-Nafs *et al.* 1990). Wound care may require early decompression, serial debridement with subsequent wound cover for salvageable limbs. Some authors advocate mandatory exploration to determine the depth and extent of injury with a view to improving limb salvage (d’ Amato, Kaplan & Brilt 1994). We, however, advocate that this be tailored to the peculiarities of each case as unnecessary explorations can increase morbidity.

Some authors have also recommended the use of nuclear imaging (Hunt *et al.* 1978) and high resolution colour and pulse doppler ultrasonography (Chen *et al.* 2003) to identify areas of muscle necrosis and injury to determine need for possible exploration.

The patients reviewed lost both upper limbs. This by any means is devastating, considering the extent of incapacitation.

The issue of cost of treatment and rehabilitation remains a heavy burden especially in our subregion where health insurance coverage is still a rarity. The only locally manufactured upper limb prostheses are of the cosmetic types which are expensive. Functional types have to be imported at exorbitant costs, often beyond the financial capacity of the victims. This underscores the need for enlightenment and prevention as most exposures are purely accidental.

**FIGURE 4:**
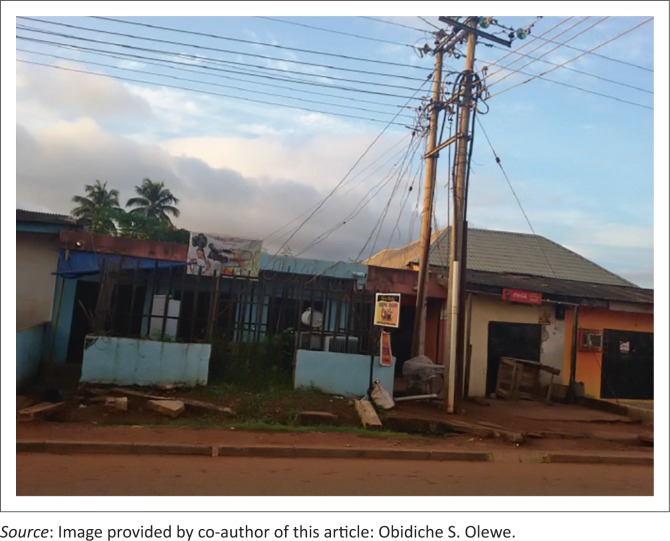
A commercial building situated less than two meters behind an 11 000 V transformer.

A common finding amongst the three patients is the unsafe proximity of the residential buildings to the overhead high voltage electrical lines and transformers – a breach of minimum clearance standards. A casual observation in our urban cities suggests many buildings are improperly situated (Figure 4), especially the high rise structures, exposing occupants to potential danger. Efforts by regulatory authorities to enforce minimum safe clearance of residential buildings as well as public enlightenment will go a long way to preventing high voltage electric injuries especially amongst non-electricity workers.
